# A prospective cohort study evaluating screening and assessment of six modifiable risk factors in HPB cancer patients and compliance to recommended prehabilitation interventions

**DOI:** 10.1186/s13741-020-00175-z

**Published:** 2021-02-17

**Authors:** Laura van Wijk, Lizzel van der Snee, Carlijn I. Buis, Judith E. K. R. Hentzen, Marjolein E. Haveman, Joost M. Klaase

**Affiliations:** Department of Hepato-Pancreato-Biliary Surgery and Liver Transplantation, University Medical Centre Groningen, PO Box 30001, 9700 RB Groningen, The Netherlands

**Keywords:** Preoperative care pathway, Screening, Risk stratification, Prehabilitation

## Abstract

**Introduction:**

Despite improvements in perioperative care, major abdominal surgery continues to be associated with significant perioperative morbidity. Accurate preoperative risk stratification and optimisation (prehabilitation) are necessary to reduce perioperative morbidity. This study evaluated the screening and assessment of modifiable risk factors amendable for prehabilitation interventions and measured the patient compliance rate with recommended interventions.

**Method:**

Between May 2019 and January 2020, patients referred to our hospital for HPB surgery were screened and assessed on six modifiable preoperative risk factors. The risk factors and screening tools used, with cutoff values, included (i) low physical fitness (a 6-min walk test < 82% of patient’s calculated norm and/or patient’s activity level not meeting the global recommendations on physical activity for health). Patients who were unfit based on the screening were assessed with a cardiopulmonary exercise test (anaerobic threshold ≤ 11 mL/kg/min); (ii) malnutrition (patient-generated subjective global assessment ≥ 4); (iii) iron-deficiency anaemia (haemoglobin < 12 g/dL for women, < 13 g/dL for men and transferrin saturation ≤ 20%); (iv) frailty (Groningen frailty indicator/Robinson frailty score ≥ 4); (v) substance use (smoking and alcohol use of > 5 units per week) and (vi) low psychological resilience (Hospital Anxiety and Depression Scale ≥ 8). Patients had a consultation with the surgeon on the same day as their screening. High-risk patients were referred for necessary interventions.

**Results:**

One hundred consecutive patients were screened at our prehabilitation outpatient clinic. The prevalence of high-risk patients per risk factor was 64% for low physical fitness, 42% for malnutrition, 32% for anaemia (in 47% due to iron deficiency), 22% for frailty, 12% for smoking, 18% for alcohol use and 21% for low psychological resilience. Of the 77 patients who were eventually scheduled for surgery, 53 (68.8%) needed at least one intervention, of whom 28 (52.8%) complied with 100% of the necessary interventions. The median (IQR) number of interventions needed in the 77 patients was 1.0 (0–2).

**Conclusion:**

It is feasible to screen and assess all patients referred for HPB cancer surgery for six modifiable risk factors. Most of the patients had at least one risk factor that could be optimised. However, compliance with the suggested interventions remains challenging.

**Supplementary Information:**

The online version contains supplementary material available at 10.1186/s13741-020-00175-z.

## Introduction

For hepatic, pancreatic and biliary (HPB) tumours, surgical resection is still the cornerstone of curative treatment. However, resections of HPB tumours are complex operations and are still associated with a high risk of complications despite improvements in perioperative care. The risk of perioperative complications depends partly on patient-related risk factors (Glance et al. 2014). However, patients’ modifiable risk factors (e.g., low physical fitness and malnutrition) also offer a good opportunity to reduce perioperative risk. The optimisation of patients’ modifiable risk factors to strengthen their resilience against the stress of surgery—a practice known as prehabilitation—is increasingly gaining ground (Silver 2015; Li et al. 2013; Hughes et al. 2019; Thomas et al. 2019; Scheede-Bergdahl et al. 2019). Although the evidence to support this practice is not yet conclusive, it is mounting rapidly. Although prehabilitation does not harm anyone, it has been suggested that a prehabilitation programme is most beneficial for high-risk patients (Barberan-Garcia et al. 2019; Berkel et al. 2018; Barberan-Garcia et al. 2018; Bongers et al. 2020).

To select high-risk patients, structured and objective screening is necessary. In the current approach to preoperative assessment, patients are referred to a (consultant) surgeon before being listed for surgery. Surgeons traditionally assess their patients’ fitness for surgery based on their own clinical judgement (Dale et al. 2014). It has been shown that this practice often results in subjective and unreliable data (Wijeysundera et al. 2018). Furthermore, to reduce perioperative risk, attention should be paid not only to patients’ physical fitness, but also to other modifiable risk factors including malnutrition, iron-deficiency anaemia, frailty, substance use and low psychological resilience (Scheede-Bergdahl et al. 2019; Bongers et al. 2020; Carli et al. 2017; Carli and Ferreira 2018).

Ideally, every patient scheduled for major surgery would undergo a structured screening and assessment based on these six risk factors. A structured screening and assessment might help in the process of shared decision-making and will select the high-risk patients who may benefit from a prehabilitation programme (Grocott 2019). To achieve this, it is necessary to reengineer the preoperative (Glance et al. 2014; Grocott et al. 2019; Grocott et al. 2017).

The aim of the present study was to evaluate the screening and assessment of modifiable risk factors amendable for prehabilitation interventions and to measure the patient compliance rate with recommended interventions. The six modifiable risk factors that were screened were low physical fitness, malnutrition, iron-deficiency anaemia, frailty, substance use (smoking/alcohol use) and low psychological resilience (stress/anxiety).

## Methods

### Study design and setting

This was a prospective cohort study that was conducted between May 2019 and January 2020. It describes the first 100 consecutive patients who followed a newly developed standard preoperative care pathway for patients referred for HPB cancer surgery at the University Medical Centre Groningen in the Netherlands. All patients completed the informed consent process, which was approved by the Institutional Review Board of the University Medical Centre Groningen (Netherlands research registration number 201800293).

### Participants

The new preoperative care pathway of the University Medical Centre Groningen was implemented for patients who met the following criteria: (i) a referral for surgical evaluation for a liver, biliary or pancreatic tumour because of (suspicion of) a malignancy; (ii) a tumour that was technically resectable, as determined by multidisciplinary consultation; (iii) age > 18 years; and (iv) ability to read Dutch.

### Preoperative care pathway

#### Original preoperative care pathway

In the original preoperative care pathway, referred patients were discussed in a multidisciplinary consultation, and if the tumour was technically resectable, the patient was invited for a consultation with the surgeon. The surgeon assessed the patient’s medical operability based on his or her own clinical judgement. If the patient was placed on the waiting list for surgery, the patient visited the anaesthesiologist in the weeks before surgery.

#### The new reengineered preoperative care pathway

**Fig. 1:**
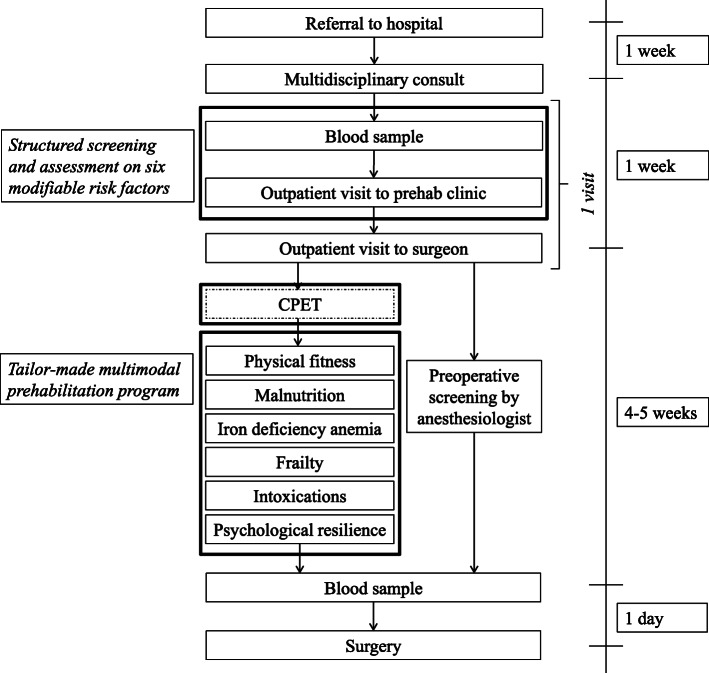
Preoperative care pathway with timeline after the implementation of the prehabilitation (outpatient) clinic; the squares outlined in bold black lines contain the new parts of the care pathway

In the new preoperative care pathway, patients first went to the prehabilitation outpatient clinic and then had an appointment with the surgeon on the same day. At the prehabilitation outpatient clinic, patients were screened and assessed for six modifiable risk factors using a structured process that included digital questionnaires, a laboratory test and functional tests. The screening occurred prior to the surgeon’s visit to enable the surgeon to include the results of the screening in the assessment of the patient’s operability. In addition, this enabled the surgeon to emphasise the importance of optimising the identified risk factors. If the patient had been listed for surgery, a tailor-made prehabilitation programme was started as soon as possible to make optimal use of the waiting time. The prehabilitation programme had no impact on the duration of the waiting period. During the waiting time, the patient consulted the anaesthesiologist, who had the information available from the structured screening and assessment (Fig. 1).

#### The prehabilitation outpatient clinic

The basic structure of the prehabilitation outpatient clinic included the following: (i) oncology nurse practitioners, who recruited all eligible patients and set up patients’ appointments and visits; (ii) a clinician (also the coordinators of the study (LW, JH and MH)) who examined the patients at the prehabilitation outpatient clinic, created a report of the test results in the electronic patient file and dealt with the referrals necessary for the interventions; and (iii) a clinician who supervised the cardiopulmonary tests. In our case, this was a sports doctor.

Patients were asked to answer digital questionnaires prior to their appointment. Laboratory tests were performed just before their visit to the prehabilitation outpatient clinic, and additional tests were conducted during the visit. The results of the laboratory tests, the prefilled digital questionnaires and functional tests were then discussed with the patient, and the recommended interventions and their importance were explained. Typically, 1 h was reserved for this appointment.

#### Digital questionnaires

The digital questionnaires were sent an average of 1 week before patients visited the prehabilitation outpatient clinic. The questionnaires included the screening tools for some of the six risk factors, together with questionnaires about patients’ highest completed educational level and work situation. Health literacy was determined using the Brief Health Literacy Screen (BHLS). The BHLS questionnaire consists of three items on a 5-point response scale. Low health literacy was identified by a score of ≥ 3 on at least one of the three questions (Sand-Jecklin and Coyle 2014). An instruction letter was enclosed with the questionnaires that contained the telephone number of the prehabilitation coordinator to enable patients to call for help if they had any queries concerning the questionnaires. In addition, the instructions contained the advice to complete the digital questionnaire with (grand)children or other relatives if necessary. Finally, if patients were not able to fill in the digital questionnaires in advance, they could do so during the prehabilitation outpatient clinic visit.

#### Screening tools for the risk factors and associated interventions

##### Physical performance

All patients referred to the clinic were screened for low physical performance. The weekly exercise activities of the patients were discussed. During their visit to the prehabilitation outpatient clinic, patients took the 6MWT, which was used to globally assess their physical performance. The following formula was used to calculate the norm value for each patient: distance = 218 + (5.14 × length [cm] − 5.32 × age) − (1.80 × weight) + (51.31 × gender [1 = male, 0 = female]) (Troosters et al. 1999). When patients scored less than 82% of their calculated norm on the 6MWT or did not comply with the global recommendations on physical activity for health (less than 2.5 h per week of moderate exercising) (World Health Organization 2010), they were referred for a cardiopulmonary exercise test (CPET) (Wasserman et al. 1999). The CPET was usually performed 1 week after the visit to the prehabilitation outpatient clinic. Patients who, after their visit to the surgeon, were not scheduled for surgery were not scheduled for a CPET. The anaerobic threshold (AT) assessed by the CPET was used to objectively determine patients’ physical fitness. An AT ≤ 11 mL/kg/min was defined as low cardiorespiratory fitness, and those patients were considered at high risk (Wilson et al. 2010). Patients with an anaerobic threshold ≤ 11 mL/kg/min were asked to participate in the PRIOR study (Netherlands Trial Register [NL6151]), a personalised home-based exercise prehabilitation programme (Berkel et al. 2020). Furthermore, patients who had an AT > 11 mL/kg/min were encouraged to follow the global recommendations on exercise (World Health Organization 2010).

##### Malnutrition

Patients’ nutritional status was determined with the Patient-Generated Subjective Global Assessment (PG-SGA) (Bauer et al. 2002). When patients scored ≥ 4 or had clinical signs of malnutrition (e.g., unexplained weight loss), they were referred to a specialised dietician. Patients who were not at risk were also made aware of the health benefits of a healthy diet, and the importance of adequate protein intake was emphasised.

##### Anaemia

Laboratory tests were already routinely conducted on each patient before they visited the surgeon. To assess whether patients suffered from iron-deficiency anaemia, ferritin, transferrin saturation (TSAT), iron and transferrin were added as standard items to the laboratory screening. Patients with an Hb-level < 12 g/dL (7.5 mmol/L) for women and < 13 g/dL (8 mmol/L) for men and with a TSAT ≤ 20% received an iron injection (Froessler et al. 2016). Iron(III)isomaltoside-1000 was used for the injection because patients undergoing liver surgery are prone to hypophosphatemia (Nomura et al. 2014), which is more often described as a side effect of ferric carboxymaltose than of iron(III)isomaltoside-1000 (Wolf et al. 2020). The exclusion criteria for receiving an iron injection were as follows: a serum ferritin level ≥ 500 μg/L, decompensated liver disease, contraindication for parenteral iron preparations and iron disorders (e.g., haemochromatosis and haemosiderosis).

##### Frailty

Patients’ frailty was measured with both the Robinson frailty score and the Groningen Frailty Indicator (GFI) (Peters et al. 2012; Robinson et al. 2013). The Robinson frailty score consists of seven frailty characteristics, each with its own cutoff values: (i) Timed Up and Go test ≥ 15 s; (ii) Katz score ≥ 1; (iii) Mini-Cog ≤ 3; (iv) Charlson Comorbidity Index ≥ 13; (v) haemoglobin ≤ 12 g/dL (7.5 mmol/L); (vi) albumin ≤ 34 g/L; and (vii) number of times the patient has fallen in the last 6 months ≥ 1. Together, these items yielded a score between 0 and 7, where a score of ≥ 4 was considered frail. The GFI is a 15-item validated questionnaire for the elderly, and the questions yielded a score between 0 and 15, where a score ≥ 4 was considered frail. Patients who were frail based on either the Robinson frailty score or GFI were referred to the geriatrician for a comprehensive geriatric assessment (Hernandez Torres and Hsu 2017).

##### Substance use (smoking/alcohol)

Patients were asked about their smoking and alcohol drinking behaviours. Preoperative smoking and motivation to quit were assessed with the following questions: ‘Do you smoke?’ ‘If so, do you intend to quit smoking?’ Preoperative alcohol (ab)use was assessed with the following questions: ‘Do you ever drink alcohol?’ ‘If so, how many glasses a week on average?’ All patients who smoked or (ab)used alcohol were strongly advised to quit. In addition, they were referred to their general practitioner for guidance to stop smoking and/or drinking alcohol (Tonnesen et al. 2010).

##### Psychological resilience (anxiety/depression)

Patients were screened for psychological resilience. The Hospital Anxiety and Depression Scale (HADS) was used to determine the need for reduction of stress and/or anxiety (Zigmond and Snaith 1983). Patients with a score ≥ 8 on one of the subscales (anxiety or depression) were advised to make an appointment with the mental health nurses who work in partnership with their own general practitioners.

In the event that a patient was advised to make an appointment with their general practitioner for guidance to stop smoking or using alcohol or for psychosocial support, their general practitioner was informed via a letter by the clinician about the advice given to the patient. However, the patient needed to make the appointment by him/herself.

#### Data collection

Baseline patient characteristics included sex, age, body mass index, American Society of Anaesthesiologists score (ASA), Charlson Comorbidity Index and race. Data on the digital questionnaires and on patients’ scores for the six risk factors were collected. Additionally, patients’ adherence to their prescribed interventions was recorded as well as their reasons if they did not adhere to one or more of the interventions.

#### Statistical analysis

Data were analyzed using SPSS for Windows (version 23.0; IBM, SPSS Inc., Chicago, IL, USA). Continuous data were presented as mean and standard deviation (SD) or as median and interquartile range (IQR) where appropriate. Categorical data were summarised by frequency and percentage.

## Results

### Baseline characteristics of the study group

We analysed the first consecutive 100 patients who had been screened at the prehabilitation outpatient clinic. All gave consent for inclusion in the study. Of these, 51 patients were men, and the median (IQR) age of all patients was 72 years (66–76). Forty-three patients had an ASA score ≥ III. The majority (77%) were eventually scheduled for surgery. Fifteen patients were not listed for surgery due to their physical status; the harm of operative intervention was expected to outweigh any potential benefits of surgery. The other eight patients were not listed for surgery because their tumours seemed benign or were judged technically irresectable after additional tests. More than half (54%) of patients were judged to have limited health literacy based on the BHLS. Finally, most of the patients (57%) were retired. More details on the basic characteristics of the participants are presented in Table 1.

**Table 1:** Baseline characteristics

Characteristics	Total 100 patients *n* = %
Sex	
Male	51%
Female	49%
Age	
18–64	21%
65–74	44%
≥ 75	35%
Median (IQR)	72 (66–76)
Body mass index	
< 18.5	1%
18.5–25	40%
25.1–29.9	35%
≥ 30	24%
Mean (SD)	27.09 (5.07)
ASA	
I–II	57%
≥ III	43%
Charlson Comorbidity Index	
< 5	20%
5–9	72%
≥ 10	8%
Mean (SD)	6 (2)
Indication for referral	
Liver tumour	24%
Biliary tract tumour	27%
Gallbladder tumour	6%
Pancreatic head tumour	31%
Pancreatic corpus/tail tumour	7%
Other	5%
Eventually scheduled for surgery	
Yes	77%
No, (partly) due to the patient’s condition	15%
No, other reason^a^	8%
Ethnicity	
Dutch	99%
Other	1%
Health literacy (BHLS) ^b^	
Low health literacy^c^	54%
Education^b^	
None	2.4%
Elementary school	9.5%
High school	39.3%
Secondary vocational	25.0%
College or university	23.8%
Main source of income^b^	
Salary	16.7%
Pension(s)	57.4%
Social allowance(s)	20.4%
None or other sources of income	5.5%

*SD* standard deviation, *BHLS* Brief Health Literacy Screen

^a^Reasons included suspicion of benign or immune-mediated tumour, irresectable before listed for surgery

^b^Data of 16 patients were missing, and thus in this case *n* = 84

^c^Identified by a score of ≥ 3 on at least one of the three questions

#### Digital questionnaires and duration of visit to the prehabilitation outpatient clinic

Despite the relatively advanced age (median 72 years; IQR 66–76) of the patients and the relatively short period of time (average 1 week) between being sent the digital questionnaires and visiting the prehabilitation outpatient clinic, 84% of all patients completed the questionnaires in advance. The reserved time of 1 h for (i) taking the tests, (ii) discussing the results and (iii) explaining the interventions was sufficient for all patients.

#### Prevalence of the risk factors and compliance with the interventions (Table 2)

##### Low physical fitness

Of the 100 patients, 64% had low physical fitness based on the 6MWT or low activity level. Forty-two of the 64 unfit patients were eventually placed on the waiting list for surgery after a visit to the prehabilitation outpatient clinic and the surgeon and were advised to perform a CPET. However, only 33 of the 42 patients performed a CPET. Four patients did not undergo a CPET due to severe knee problems; five others could not have a CPET (re)scheduled because they had intermittent hospital admissions due to cholangitis (2 patients) or portal vein embolisation (1), or because they missed their initial CPET appointment due to another appointment at the hospital taking longer than expected (2). Of the patients who did undergo a CPET, 17 (52%) had an AT ≤ 11 mL/kg/min; among them, 15 were requested to enrol in the PRIOR study (preoperative home prehabilitation for patients planned for pancreatic or liver resection) (Berkel et al. 2020). One patient ended up undergoing radiofrequency ablation, which is an exclusion criterion for the PRIOR study. In another patient, surgeons preferred short-term surgery because of borderline resectability. Eventually, 12 out of the 15 patients (80%) were included in the PRIOR study. In two cases, we could not find an available physical therapist to supervise the patient, and in one case, no home trainer was available because all were already occupied. Results of the PRIOR study will be published separately.

**Table 2:** Results of the screening, assessments and interventions

Risk factor	Total screened	Unfit (6 MWT–activity level)	CPET advised	CPET performed	Unfit (AT ≤ 11 ml/kg/min)	PRIOR advised	PRIOR complied
Physical fitness	**100**	**64**	**42**^**a**^	**33**	**17**	**15** ^**b**^	**12**
	Total screened	Malnourished	Dietician advised	Dietician complied			
Malnutrition	**100**	**42**	**23** ^**c**^	**19**			
	Total screened	Anaemia	Iron deficiency	Iron therapy prescribed	Iron therapy administered		
Anaemia	**100**	**32**	**15**	**12**^**d**^	**12**		
	Total screened	Frail	Geriatrician advised	Geriatrician complied			
Frailty	**100**	**22**	**12**^**e**^	**12**			
	Total screened	Smoking	Intervention advised	Intervention complied			
Smoking	**100**	**12**	**9**^**f**^	**1**			
	Total screened	Alcohol (ab)use (> 5 units per day)	Intervention advised	Intervention complied			
Alcohol use	**100**	**18**	**14**^**g**^	**8**			
	Total screened	Low psychological resilience	Psychological help advised	Psychological help complied			
Low psychological resilience	**100**	**21**	**14**^**h**^	**4**			

^a^Eventually not scheduled for surgery (*n* = 22)

^b^Preference for surgery on short-term (*n* = 1). Exclusion criteria PRIOR study (*n* = 1)

^c^Eventually not scheduled for surgery (*n* = 12). Already under treatment (*n* = 7)

^d^Eventually not scheduled for surgery (*n* = 2). Already under treatment (*n* = 1)

^e^Eventually not scheduled for surgery (*n* = 10)

^f^Eventually not scheduled for surgery (*n* = 3)

^g^Eventually not scheduled for surgery (*n* = 4)

^h^Eventually not scheduled for surgery (*n* = 6). Already under treatment (*n* = 1)

##### Malnutrition

Forty-two (42%) patients were at risk of malnutrition based on the PG-SGA. Twelve patients eventually did not undergo surgery, and seven patients were already under treatment from the dietician on the advice of the referring hospital. The remaining 23 patients were referred to a primary or secondary care dietician, of whom 19 (82%) patients did visit a dietician. The other four patients did not visit the dietician because they did not see the additional benefit of it.

##### (Iron-deficiency) anaemia

Among all the patients, 32 (32%) suffered from anaemia, which was caused by iron deficiency in 15 (47%) of them. Of those patients with an iron deficiency, 13 were eventually scheduled for surgery. Of these 13, one patient was already receiving oral iron supplementation, which had been prescribed by the referring specialist. We prescribed an iron injection for 12 patients, and all 12 were administered.

##### Frailty

Of all patients, 22 (22%) were frail based on the GFI (*n* = 22) and Robinson frailty scores (*n* = 7). Twelve of these patients were eventually scheduled for surgery and were therefore referred for a comprehensive geriatric assessment supervised by the geriatrician. The advice given by the geriatrician included delirium prevention and optimisation of drug treatments for comorbidities such as heart failure and diabetes. Attention was also paid to patients’ social networks.

##### Substance use (smoking/alcohol)

Of all patients, 12 (12%) reported that they smoke. Nine of these patients were eventually scheduled for surgery and were strongly encouraged to stop smoking. These patients were subsequently advised to visit the general practitioner for guidance on smoking cessation. Four of these nine patients indicated that they planned to stop, two patients indicated that they were motivated to quit but lacked confidence that they would succeed, and three patients had no intention to stop smoking. Only one of the nine patients (11%) was able to quit smoking with help from her general practitioner. The other eight patients failed to stop smoking with or without the help of their general practitioner. Eighteen (18%) patients said they consumed more than five units of alcohol per week, but only four of them said they drank more than 21 units per week, which is the level of alcohol consumption regarded as hazardous by to the World Health Organization. Fourteen patients were eventually scheduled for surgery and were strongly advised to stop drinking alcohol. Eight of the 14 (57%) were able to stop; however, only one of these eight drank more than 10 units of alcohol per week before the assessment. Six patients did not stop drinking alcohol because they were not sufficiently motivated to stop. Five of these six patients drank more than 10 units per week, of whom one patient drank more than 21 units per week.

##### Low psychological resilience

The HADS indicated the presence of anxiety or depression disorders in 21 (21%) of the patients. Fifteen patients were eventually scheduled for surgery, of whom one already had psychological help. Only four of the other 14 (29%) sought psychological help after we advised them to do so. The other ten patients were not interested in receiving psychological help.

#### Compliance with the interventions

The median (IQR) number of interventions needed in the 77 patients scheduled for surgery was 1.0 (0–2). Of the patients who were scheduled for surgery, only 24 (31.2%) had no risk factors and therefore did not require an intervention. Of the 53 patients who needed one or more interventions, 28 (52.8%) complied with 100% of the necessary interventions. The percentage of patients who complied with 100% of the necessary interventions diminished as patients needed more interventions. However, the number of patients with three or more risk factors was very small, so these results should be interpreted with caution. More details can be found in Table 3.

**Table 3:** Distribution of necessary interventions per patient and the mean compliance with all of the interventions

Number of necessary interventions	Patients scheduled for surgery *n* = 77, *n* (%)	% patients compliant with all interventions, *n* (%)
0	24 (31.2%)	Not applicable
1	25 (32.5)	16 (64.0%)
2	17 (22.1%)	9 (52.9%)
3	6 (7.8%)	2 (33.3%)
4	4 (5.2%)	1 (25.0%)
5	0 (0.0%)	Not applicable
6	1 (1.3%)	0 (0.0%)
7	0 (0.0%)	Not applicable

Interventions include (1) inclusion in PRIOR study, (2) consultation with dietician, (3) iron injection, (4) assessment by geriatrician, (5) cessation of smoking with help from general practitioner, (6) cessation of alcohol use with help from general practitioner and (7) psychosocial help

#### Differences in number of risk factors and compliance to interventions between low and normal health literacy

Patients with a low literacy (*n* = 45) had a significant (*P* = 0.021) higher median number of risk factors 2 [IQR; 1–3] than patients with no limited health literacy (*n* = 39) 1 [IQR; 0–2]. We also found a significant difference between the two groups when only the patients that were scheduled for surgery were compared (*P =* 0.020). We found no significant association between health literacy and compliance to their interventions (*P* = 0.278). However, we did find an interesting outcome when comparing just the percentage of patients who completed 100% of the interventions within both groups. The percentage of patients that complied to all of the interventions was 45.8% in the low literacy group versus 70.6% in the normal literacy group.

## Discussion

The aim of this study was to evaluate the screening and assessment of modifiable risk factors amendable for prehabilitation interventions and to measure the patient compliance rate with recommended interventions for oncological HPB patients. Our results indicate that it is feasible to implement a structured screening and assessment for six modifiable risk factors by reengineering the preoperative care pathway. Two thirds of our referred patients who were eventually scheduled for HPB cancer surgery needed preoperative optimisation of one or more risk factors. Compliance with all of the necessary interventions was nearly 53%, indicating that there is room for improvement.

The preoperative process has two important functions: (1) to ensure that the patient is as prepared as possible to withstand the stress of surgery (i.e., prehabilitation) and (2) to ensure an optimal process for shared decision making about whether to perform surgery (Grocott 2019). In this study, the prehabilitation screening was implemented before the patients were seen by the surgeon. Early engagement with patients not only creates a longer window for prehabilitation but also adds value to the surgeon’s assessment of medical operability and consequent informed shared decision-making. By contrast, other studies in the field of prehabilitation scheduled their prehabilitation screening when it was already decided that the patient would undergo surgery (Barberan-Garcia et al. 2018; van Rooijen et al. 2019; Carli et al. 2020).

Surgeons in this study experienced the prehabilitation outpatient clinic and the timing of screening positively. They found the objective test results of the screening at the prehabilitation outpatient clinic of additional value in their assessment of operability. Through this accurate preoperative risk screening, we have tried to avoid operating on patients for whom the harm of operative interventions is expected to outweigh any potential benefits of surgery. At the same time, patients who could benefit the most from a prehabilitation programme were selected. The surgeons in our study also indicated that it was useful for them to know which risk factors applied to the patients, which enabled them to pay specific attention to these factors during their consultations.

While the field of prehabilitation is beginning to adopt a multimodal approach, this is the first study that evaluates the implementation of a structured programme to optimise oncological HPB patients after screening and assessment for six modifiable risk factors (Scheede-Bergdahl et al. 2019; van Rooijen et al. 2019; Carli et al. 2020; Luther et al. 2018). Currently, little evidence exists for patients’ willingness to address multiple behaviours simultaneously in the prehabilitation context (Luther et al. 2018; McDonald et al. 2019). McDonald and colleagues explored the effects of motivation for, confidence about and priority of changing health behaviours before surgery on short-term perioperative health benefits compared with long-term general health benefits. They concluded that patients exhibited favourable attitudes towards changing single and multiple health behaviours before surgery (McDonald et al. 2019). In our study, we observed a large degree of variation in compliance with the interventions, which was also described in a review by Luther et al. (Luther et al. 2018). In spite of the fact that patients exhibited a favourable attitude towards all interventions during the consultations in the prehabilitation outpatient clinic, compliance was highly dependent on the type of intervention.

Compliance with performing a CPET was 87%. However, this was not caused by a lack of willingness on the part of the patients but rather was due to limited availability of the CPET and medical problems (e.g., osteoarthritis of the knee). In an earlier study by Wilson et al. (Wilson et al. 2010), patients underwent a CPET as part of preoperative screening for colorectal surgery, bladder or kidney cancer surgery, and 54% of patients had an AT below 10.9 mL/kg/min. We found a similar percentage (52%) in our study; however, we had already preselected patients for a CPET, which was not the case in the study of Wilson et al. (except for patients aged > 55 years). The results of the PRIOR study are not yet available, as the inclusion was completed in April 2020. However, preliminary results are promising (Van Wijk et al. 2020), and results are expected soon. In our study, 42% of patients were at risk of malnutrition, whereas La Torre et al. reported 53% among patients scheduled for pancreatic surgery (La Torre et al. 2013). Another study screened all patients admitted to hospital and reported that 47% were malnourished (Jeejeebhoy et al. 2015). In our study, 82% of the referred patients visited the dietician. In another study, compliance with nutritional therapy was reported to be between 72 and 96.6% (Luther et al. 2018).

In addition, we found that 32% of patients suffered from anaemia, and in 47% of these patients, the anaemia was caused by an iron deficiency. Munoz et al. reported the presence of preoperative anaemia in 36% of patients undergoing major abdominal surgery, with an absolute iron deficiency in 61.25% of cases (Munoz et al. 2017). This difference in incidence rates could be explained by differences in the type of underlying diseases from which patients suffer.

In previous studies, the presence of frailty varied between different types of surgery and assessment tools, range between 10.4 and 37.0% (Hewitt et al. 2018). We reported 22% in the present study. All patients who were referred to the geriatrician or for an iron injection attended their appointment.

The HADS indicated the presence of an anxiety or depression disorder in 21% of all patients in our study; this is comparable with the results of Clark et al., who found depression or anxiety in 29% in patients with pancreatic cancer (Clark et al. 2010). In our study, only 29% sought psychological help. In a review of Mosher et al. about psychosocial interventions for patients with colorectal cancer, compliance varied between 62 and 97%; however, interventions were better facilitated and supplied than in our study (Mosher et al. 2017).

In our study, 12% of the patients smoked, and 18% consumed more than 5 units of alcohol per week. These percentages were lower than those reported for the incidence of smoking (30%) in patients scheduled for general surgery but comparable with the 7–49% incidence of hazardous drinking (Tonnesen et al. 2009). In our study, only 11% of patients were able to stop smoking, which is lower than the percentages reported in relevant literature, which have varied from 35 to 89% depending on the type of intervention (Tonnesen et al. 2009; Boylan et al. 2019). In our study, more patients (57%) managed to quit alcohol, which is in line with the results of McDonald et al. who reported patients being more motivated and confident about quitting alcohol than smoking (McDonald et al. 2019). Despite the modest success rates for quitting smoking and alcohol in this study, the results showed that even a brief intervention (giving advice to quit and seeking help from the patient’s general practitioner if necessary) can be beneficial. However, to achieve optimal results for preoperative smoking and alcohol control interventions, intensive programmes with competent and dedicated health professionals are required (Tonnesen et al. 2009).

Overall, compliance with hospital appointments was higher than in interventions where the initiative for making the appointment rested with the patient. These results underline both the usefulness of and the need for intensive support services to help patients with optimising their modifiable risk factors (McDonald et al. 2019). This suggests that it is important to include preoperative risk stratification and prehabilitation as an integral part of the perioperative care package.

An important strength of our study was that the screening consisted of six potential risk factors and that the preoperative screening was already incorporated in the perioperative care pathway. An important limitation of the study is that we did not investigate the effect of the interventions. In order to evaluate the effect of all interventions, patients would have to be re-evaluated after the intervention, which would be logistically difficult. Moreover, the actual effect of interventions is difficult to determine due to side effects of interventions on the different domains. In addition, the study was conducted in a specific population in a university hospital, which reduced the generalizability of the results. Because not all interventions carried out by the patient could be verified (e.g., a patient who told that he had visited a dietician), the results should be interpreted with caution. It is clear that multimodal prehabilitation is likely to improve postoperative outcomes (Hughes et al. 2019; Kamarajah et al. 2020). The time has now come to determine how prehabilitation should be incorporated into the preoperative care pathway. A recently published article argued that multimodal prehabilitation should include exercise, nutrition and psychosocial care (Kamarajah et al. 2020). The authors consider it a lost opportunity not to include the other known risk factors and suggest that each patient should be screened for the six risk factors as discussed in the present study, which also corresponds to the recommendation of Bongers and colleagues (Bongers et al. 2020). In addition, our results showed that 53 of the 77 HPB patients (68.8%) needed at least one intervention, and patients with a low literacy had on average significant more risk factors than patients with a normal health literacy. Furthermore, structured screening and assessment might improve the cost-effectiveness of prehabilitation programmes because prehabilitation is most beneficial for high-risk patients (Barberan-Garcia et al. 2019; Berkel et al. 2018; Barberan-Garcia et al. 2018; Goncalves and Groth 2019; Minnella et al. 2016). However, literature regarding the cost-effectiveness of prehabilitation is limited (Barberan-Garcia et al. 2019; Nielsen et al. 2008). It would be of interest to investigate the potential financial benefits of prehabilitation programmes; demonstrating such benefits might accelerate their implementation.

In conclusion, our study evaluated the implementation of a prehabilitation outpatient clinic for patients referred for hepatobiliary and pancreatic cancer surgery. It was feasible to reengineer the preoperative care pathway in order to implement a structured screening and assessment for six modifiable risk factors before the patients visited the surgeon. Especially when the initiative for the intervention lies with the patient, it is a challenge to ensure that all patients adhere to their necessary interventions during the waiting period. To ensure patients comply with all interventions, intensive programmes with competent and dedicated health professionals are required.

## Supplementary Information

**Additional file 1.**

## Data Availability

Data are available from the corresponding author on reasonable request.

## Abbreviations

ASA: American Society of Anaesthesiologists score
BHLS: Brief Health Literacy Screen
CPET: Cardiopulmonary exercise test
GFI: Groningen Frailty Indicator
HADS: Hospital Anxiety and Depression Scale
HPB: Hepatic, pancreatic and biliary
PG-SGA: Patient-Generated Subjective Global Assessment
TSAT: Transferrin saturation
6MWT: 6-minute walk test
