# Supplementary material for: A prospective cohort study evaluating screening and assessment of six modifiable risk factors in HPB cancer patients and compliance to recommended prehabilitation interventions
Source: Perioper Med (Lond). 2021 Feb 17;10:5. doi: 10.1186/s13741-020-00175-z (PMC7887817; doi:10.1186/s13741-020-00175-z)
Supplement: Supplementary file 1 — Additional file 1. [file 13741_2020_175_MOESM1_ESM.docx]

Supplementary file

| **1.** Cardio Pulmonary Exercise Test | |
| --- | --- |
| ***Anaerobic Threshold (ml/kg/min)*** | **Total n = 33 (100%)** |
| 8.0 – 9.0 | 7 (21.2%) |
| 9.1 – 10.0 | 3 (9.1%) |
| 10.1 – 11.0 | 7 (21.2%) |
| 11.1 – 12.0 | 5 (12.2%) |
| 12.1– 13.0 | 6 (18.2%) |
| 13.1 – 14.0 | 1 (3.0%) |
| ≥14.1 | 4 (12.1%) |

| **2.** Patient Generated Subjective Global Assessment | |
| --- | --- |
| ***Score*** | **Total n = 100 = %** |
| 0 – 3 | 58 |
| 4 – 8 | 22 |
| ≥9 | 20 |

| **3.** Haemoglobin | |
| --- | --- |
| ***g/dL*** | **Total n = 100 = %** |
| ≤ 10.0 | 4 |
| 10.1 – 11.0 | 12 |
| 11.1 – 12.0 | 10 |
| 12.1– 13.0 | 22 |
| ≥13 | 52 |

| **4.** Iron status of patients with anaemia | |
| --- | --- |
| ***Transferrin saturation %*** | **Total n = 32 (100%)** |
| ≤ 10 | 2 (6.3) |
| 11 – 20 | 10 (31.3) |
| 21 – 30 | 10 (31.3) |
| 31 – 40 | 3 (9.4) |
| ≥ 41 | 7 (21.9) |

| **4.** Frailty | | |
| --- | --- | --- |
| ***Score*** | *Groningen Frailty Indicator*  **Total n = 100 = %** | *Robinson Frailty Score*  **Total n = 100 = %** |
| ≤1 | 23 | 57 |
| 2 | 16 | 22 |
| 3 | 39 | 14 |
| 4 | 5 | 5 |
| 5 | 3 | 1 |
| 6 | 4 | 0 |
| ≥7 | 10 | 1 |

| **5.** Smoking | |
| --- | --- |
| ***Number cigarettes/cigars per day*** | **Total n = 100 = %** |
| 0 | 88 |
| 1-5 | 3 |
| 5-10 | 2 |
| 10-20 | 5 |
| ≥21 | 2 |

| **6.** Alcohol use | |
| --- | --- |
| ***Units alcohol per week*** | **Total n = 100 = %** |
| 0 | 47 |
| 1-5 | 35 |
| 6-10 | 7 |
| 11-15 | 2 |
| 16-20 | 5 |
| 21-24 | 3 |
| ≥25 | 1 |

| 7. Hospital Anxiety and Depression Scale | | |
| --- | --- | --- |
| **Score** | *Anxiety*  **Total n = 100 = %** | *Depression*  **Total n = 100 = %** |
| ≤1 | 28 | 39 |
| 2-3 | 24 | 16 |
| 4-5 | 16 | 17 |
| 6-7 | 18 | 14 |
| 8-9 | 6 | 5 |
| ≥10 | 8 | 9 |
|  |  |  |
